# Cases from Practice

**Published:** 1891-05

**Authors:** 

**Affiliations:** Leipzig


### CASES FROM PRACTICE.

Dr. Lorbacher, Leipzig.

A. N. Z., 2, 1891.

1. A rachitic girl of seven years suffered from carious ulcers of the sternum, the right knee swollen, with nearly perfect flexion of the leg, so that she could only crawl or limp on one foot, though perfect anchylosis has not yet set in. She was emaciated, marantic, and ill-humored. Treatment began with Silicea $^{30}$, some globules at first daily and then more rarely, followed by Calcarea-carb. in the same manner. The ulcers at the sternum healed first, then the swelling of the knee decreased, the joint became more mobile, so that the leg could be stretched, and the atrophy of the muscles gradually gave way to a more normal state. She runs and jumps now, and her natural good humor has returned. No external adjuvants were used.

(Calcarea-silicata is with S. L. a favorite prescription in similar cases. I usually give it in the middle potencies 2 C to 5 C (200 to 500) and so far am satisfied with the results. It did me good service in some cases of hip-diseases. Saccharum-lactis is a great aid in such cases and far too much neglected.)

2. A six-months babe, of a rachitic family, and artificially brought up, was attacked by cholera infantum. Her physician prescribed Arsenicum low in frequent doses. On the fifth day Lorbacher was called in and found hydrocephaloid. The child looked pale, with sunken eyes, slightly soporous, moaning, sometimes starting up and twitching in all extremities, fontanelles sunken in, filiform pulse, chills alternating with heat, restlessness. Schweikert recommends in such cases Phosphor. and Zinc in alternation, but on account of the rachitic constitution plus the cerebral symptoms, which are also found under Sulphur, Lorbacher decided to rely on Sulphur $^{30}$, four globules every four hours. After twelve hours amelioration began, diarrhœa ceased, and the stools took on a better color and consistency, all cerebral symptoms ceased and after five days convalescence was established. The child has since off and on taken a dose of Calcarea, which aids him over the difficult crisis of dentition.

(The rachitic constitution certainly belonged to the totality of symptoms, worse by the artificial food, and it shows again and again that symptom-covering must be well understood to get successful results, and we often fail because we consider too much outward symptoms and neglect the individuality of the patient. Instead of increasing or diminishing our materia medica, let us prayerfully study, morning and evening, our antipsorics, for in them often our salvation lies.

3. In selecting the simile the sides of the body are often of great importance. He treated two left-sided cases of tonsillar angina; one of them showed a diphtheritic coating and difficulty in swallowing from relaxation of the muscles of deglutition. Lachesis cured them in a few days.

4. Kallanbach, of Rotterdam, reports the case of hystero-epilepsy major, which was given up as incurable by many physicians. The miss of twenty years was delicate and given to nervous twitchings, menstruation regular, but tardy, often complaining of headache and toothache. Sitting at the window she became *terribly frightened* by seeing children fall out of a swing, and unconscious convulsions followed, interrupted by visions of terrible accidents. Sometimes there seemed to be a little interval, but then the convulsions reappeared with full force, with the addition of vomiturition and mucous vomiting several times a day, and a total paralysis of the vocal organs. Opisthotonos, emprosthotonos, and frightful contortions alternated, and the treatment failed even to alleviate. She was emaciated to a skeleton when Kallenbach saw her, and he tried at first to gain her confidence and set her will-power in action again, for only by this mental influence he hoped to benefit her, for gradually the fits became more rare and less terrible. Her brother had to start for America, and *this depressing mental emotion* caused her to utter again her first words, " Henry is gone," and speech remained henceforth unmolested. What did cure her? asks honest Kallenbach, for after a year's treatment she is now well and blooming. It is true, regulation of the diet, encouraging oppressed and suppressed will-power to regain its energy, massage, and rubbing were faithfully followed out. Many remedies were given, as Pulsatilla, Ignatia, Plumbum, Phosphorus, Argentum, Nitre, Lycopodium, Natrum-mur., but only from Tarentula-hispanica benefit could be seen, and Kallenbach is sure that it aided greatly in curing the case. (Allen's *Handbook* gives the symptoms of Tarentula so clearly that we are convinced of its being at least a simile.) Yet the beauty of the case lies in the soul. A mental fright set the nervous system agog as it worked in an irregular, zigzag fashion, a mental depression removed the irregularity of the nerves and thus helped the Tarentula to restore its equilibrium. S. L.

